# Supplementary material for: Signatures of Admixture and Genetic Uniqueness in the Autochthonous Greek Black Pig Breed Deduced from Gene Polymorphisms Affecting Domestication-Derived Traits
Source: Animals (Basel). 2023 May 26;13(11):1763. doi: 10.3390/ani13111763 (PMC10251807; doi:10.3390/ani13111763)
Supplement: Supplementary file 1 [file animals-13-01763-s001.zip › animals-2367653-supplementary.pdf]

animals

MDPI

Supplementary Material

Table S1. PCR primers and protocols used for the genotyping of markers at the *MC1R*, *KIT*, *NR6A1*, *VRTN* and *IGF2* genes.

Primer pair names/genes	Sequence of the forward and *reverse* primers (5′-3′)	Analysis[1]	Amplified region (bp)	Annealing temperature (°C)	Genotyping protocol/system[2]
MC1R_1	CTGCACTCGCCCATGTACTA<br>*AGCAGAGGCTGGACACCAT*	PCR-RFLP	196	60	Amplicons digested with *Bsp*HI (c.367G = 196 bp in E^{+}, E^{D1} and *e*; c.367A = 154 + 42 bp in E^{D2} and E^{P}) *
MC1R_2	GCGGGTACTGTACGTCCACAT<br>*CCCAGCAGAGGAGGAAGAC*	PCR-RFLP	154	60	Amplicons digested with *Hha*I (c.727G = 108 + 46 bp in E^{+}, E^{D1}, E^{D2} and E^{P}; c.727A = 154 bp in *e*); Amplicons digested with *Bst*UI (c.729G = 109 + 47 bp in E^{+}, E^{D2} and E^{P}; c.729A = 154 bp in E^{D1} and *e*) *
MC1R_indel	CACCTCTGGGAGCCATGA<br>*GTCTGGTTGGTCTGGTTG*	Fragment analysis	168/170	55	Amplicons analyzed in a capillary sequencer (ABI3100 Avant, ABI Prism)
MC1R_seq1	CACCTCTGGGAGCCATGA<br>*AGCAGAGGCTGGACACCAT*	Sanger sequencing	479	59	Amplicons sequenced with ABI3100 Avant, ABI Prism
MC1R_seq2	GTCATGGACGTGCTCATCTG<br>*CCCAGCAGAGGAGGAAGAC*	Sanger sequencing	409	60	Amplicons sequenced with ABI3100 Avant, ABI Prism
MC1R_seq3	CACCCTCACCATCCTGCT<br>*CCCAGCCACGTAGGAACC*	Sanger sequencing	311	59	Amplicons sequenced with ABI3100 Avant, ABI Prism
KIT	TGAACATTGCTGACTCCCCT<br>*TGCATTTTACCTAAAGAGAAGAGC*	PCR-RFLP	157	56	Amplicons digested with *Dde*I (g.8:43597545C = 157 bp; g.8:43597545T = 93 + 64 bp) *
NR6A1	GGTATCCTGAGCACCCAGTC<br>*ACCTGGAGGACAGTGTGGAG*	PCR-RFLP	203	58	Amplicons digested with *Msp*I (g.299084751C = 180 + 23 bp; g.299084751T = 203 bp)*
VRTN	GGCAGGGAAGGTGTTTGTTA<br>*GACTGGCCTCTGTCCCTTG*	PCR-RFLP	411/120	56	Amplicons separated in a 2.5% agarose gel electrophoresis (allele Q = 411 bp; allele wild type (WT) = 120 bp)
IGF2	CCGCGGCTTCGCCTAGGGTC<br>*CCGGCTGGAAGGGAGGAAGC*	PCR-RFLP	172	65	Amplicons digested with *Tsp*45I (g.3072G = 172 bp; g.3072A = 155 + 17 bp) *

[1] Detailed protocols of the PCR analyses. All amplification reactions were carried out in a 25 μL total volume containing 100 ng genomic DNA, 10 pmol of each primer and the GoTaq® Hot Start Colorless Master Mix (Promega Corporation, Madison, Wisconsin, USA). A SimpliAmp thermal cycler (Thermo Fisher Scientific, Carlsbad, CA, USA) was used with the following PCR temperature profile: initial denaturation step of 5 min at 95 °C, then 35 cycles of 30 sec at 95 °C, 30 sec at 56-60°C (according to the PCR product; see this table), 30 sec at 72 °C and a final elongation step of 5 min at 72 °C. Electrophoresis on 2.5-3.5% agarose gels was carried out to detected amplified fragments and restriction enzyme digested patterns after visualized with 1× GelRed Nucleic Acid Gel Stain (Biotium Inc., Hayward, CA, USA). The c.67insCC indel of the *MC1R* gene was analyzed using a fragment analysis protocol in a 4-capillary sequencer (ABI3100 Avant, ABI Prism, Thermo Fisher Scientific, Carlsbad, CA, USA) to detect 6-FAM fluorescent amplicons. Moreover, Sanger sequencing was applied to confirm genotyping results from several amplicons for each gene, following the protocols already described [26,27].

[2] When an asterisk (*) was indicated, the genotyping protocol was based on PCR-RFLP analysis. Amplified fragments have been digested with the indicated restriction enzymes and the generated fragments after digestion for the different alleles are reported.

Table S2. Allele frequencies of the analyzed gene polymorphisms in the European pig breeds, including the Greek Black Pig breed, and wild boar populations. Information for the Greek Black Pig breed derives from the genotyping activities reported in this study. For some breeds/gene markers, information derives from previous studies [9,13,26,27,32,33,36]. When this information was not available, for some breeds and the following markers at the *NR6A1*, *VRTN* and *KIT* genes, allele frequencies derive from the mining of whole genome sequencing data produced by Bovo et al. [11].

Breeds/population	No. of pigs	*NR6A1*	*VRTN*	*IGF2*	*KIT*	*MC1R*						
		T	*Q*	*A*	*T*	*MC1R*1*	*MC1R*2*	*MC1R*3*	*MC1R*4*	*MC1R*6*	*MC1R*7*	*MC1R*10*
**Greek Black**	59	0.839	0.449	0.856	0.017	0.076	0.008	0.703	0.153	0.017	0.000	0.042
**Alentejana**	35	0.919	0.171	0.000	0.000	0.000	0.010	0.090	0.000	0.660	0.240	0.000
**Apulo Calabrese**	35	0.703	0.565	0.850	0.029	0.000	0.000	0.900	0.000	0.100	0.000	0.000
**Basque**	30	1.000	0.903	0.000	0.108	0.000	0.010	0.980	0.000	0.010	0.000	0.000
**Bísara**	35	1.000	0.321	0.010	0.000	0.000	0.000	0.860	0.010	0.130	0.000	0.000
**Black Slavonian**	35	0.865	0.432	0.080	0.037	0.000	0.890	0.040	0.020	0.060	0.000	0.000
**Casertana**	35	0.950	0.314	0.030	0.059	0.000	0.000	0.890	0.020	0.090	0.000	0.000
**Cinta Senese**	35	1.000	0.422	0.120	0.969	0.000	0.000	0.780	0.060	0.160	0.000	0.000
**Italian Duroc**	30	1.000	0.596	1.000	0.000	0.000	0.000	0.000	1.000	0.000	0.000	0.000
**Gascon**	30	1.000	0.500	0.000	0.000	0.000	0.000	0.980	0.000	0.020	0.000	0.000
**Krškopolje**	35	1.000	0.683	0.390	0.027	0.000	0.000	0.820	0.020	0.160	0.000	0.000
**Italian Landrace**	44	1.000	0.512	0.680	0.000	0.000	0.000	0.000	0.000	1.000	0.000	0.000
**Italian Large White**	49	1.000	0.400	0.912	0.011	0.000	0.000	0.000	0.000	1.000	0.000	0.000
**Lithuanian White Old Type**	35	1.000	0.553	0.100	0.000	0.000	0.000	0.000	0.000	1.000	0.000	0.000
**Lithuanian Indigenous Wattle**	35	0.500	0.400	0.280	0.000	0.000	0.000	0.000	0.130	0.870	0.000	0.000
**Swallow Bellied Mangalitsa**	35	0.975	0.438	0.000	0.750	1.000	0.000	0.000	0.000	0.000	0.000	0.000
**Mora Romagnola**	35	1.000	0.860	0.100	0.000	0.520	0.000	0.000	0.480	0.000	0.000	0.000
**Moravka**	35	0.976	0.615	0.260	0.214	0.030	0.370	0.430	0.000	0.170	0.000	0.000
**Majorcan Black**	35	0.938	0.170	0.190	0.174	0.000	0.020	0.920	0.010	0.050	0.000	0.000
**Nero Siciliano**	35	0.714	0.250	0.200	0.051	0.010	0.040	0.750	0.030	0.180	0.000	0.000
**Sarda**	35	1.000	0.543	0.270	0.068	0.010	0.040	0.260	0.030	0.660	0.000	0.000
**Schwäbisch Hällisches Schwein**	35	1.000	1.000	0.500	0.095	0.000	0.160	0.840	0.000	0.000	0.000	0.000
**Turopolje**	35	0.457	0.000	0.000	0.000	0.000	0.000	0.000	0.000	1.000	0.000	0.000
**Wild Boar**	35	0.000	0.000	0.000	0.000	1.000	0.000	0.000	0.000	0.000	0.000	0.000
